# Supplementary material for: Complex electronic structure and compositing effect in high performance thermoelectric BiCuSeO
Source: Nat Commun. 2019 Jun 27;10:2814. doi: 10.1038/s41467-019-10476-7 (PMC6597697; doi:10.1038/s41467-019-10476-7)
Supplement: Supplementary file 1 — Supplementary Information [file 41467_2019_10476_MOESM1_ESM.pdf]

Ren *et al.*

Supplementary Information

Complex electronic structure and compositing effect in high performance thermoelectric BiCuSeO

Guang-Kun Ren[1, 2, 3], Shanyu Wang[2], Zhifang Zhou[1], Xin Li[4], Jiong Yang[4], Wenqing Zhang[4], Yuan-Hua Lin[1*], Jihui Yang[2*], Ce-Wen Nan[1]

[1] State Key Laboratory of New Ceramics and Fine Processing, School of Materials Science and Engineering, Tsinghua University, Beijing, 100084, P. R. China

[2] Materials Science and Engineering Department, University of Washington, Seattle, Washington, 98195, USA

[3] Institute of Materials, China Academy of Engineering Physics, Jiangyou, Sichuan, 621908, P. R. China

[4] Materials Genome Institute, Shanghai University, Shanghai, 200333, P. R. China

Correspondence and requests for materials should be addressed to Prof. Jihui Yang (email: jihuiy@uw.edu), or to Prof. Yuan-Hua Lin (email: linyh@mail.tsinghua.edu.cn).

Supplementary Figures

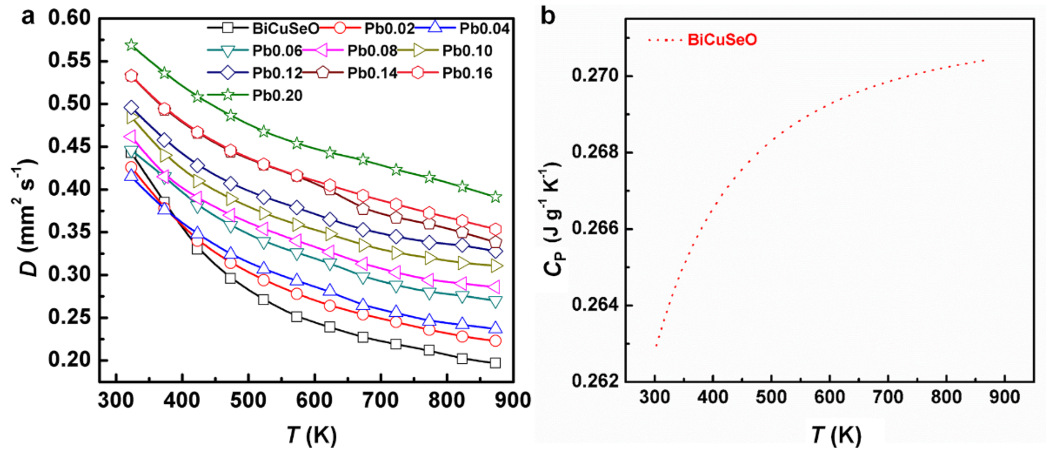

Supplementary Figure 1. Thermal transport properties of $Bi_{1-x}Pb_xCuSeO$ (x = 0-0.2). **a** The measured thermal diffusivity and **b** specific heat calculated by the Debye model (similar to the Dulong-Petit law for $T > \Theta_D$, Θ_D is the Debye temperature).

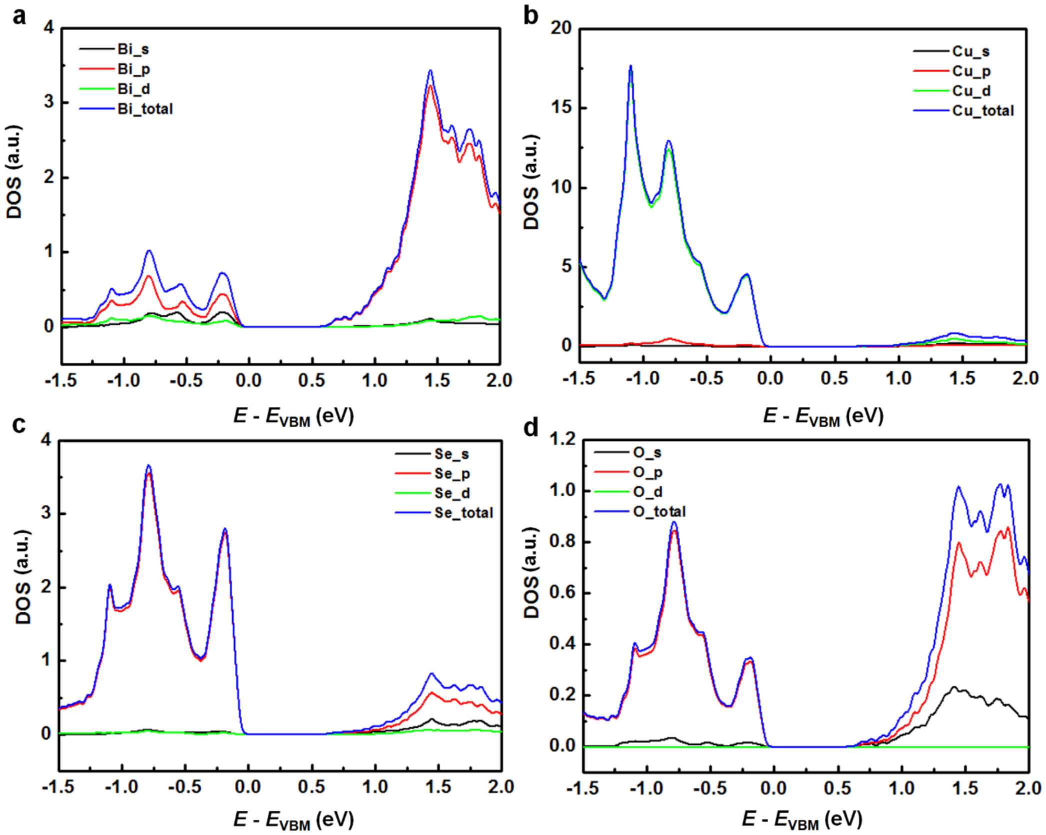

Supplementary Figure 2. The partial density of states (pDOS) of different elements for the pristine BiCuSeO. The electronic density of states (DOS) of **a** Bi, **b** Cu, **c** Se, and **d** O.

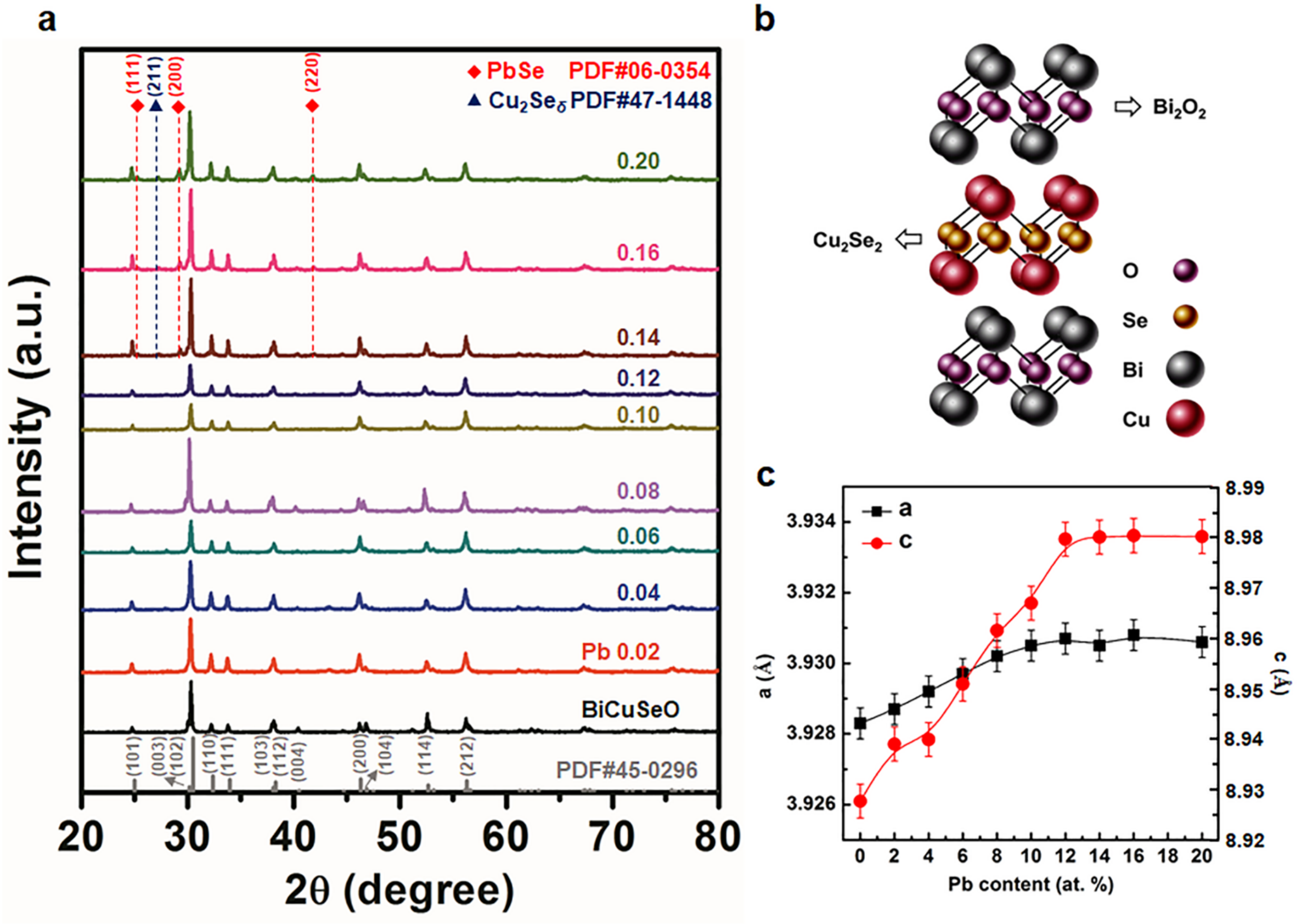

Supplementary Figure 3. Phase compositions of $Bi_{1-x}Pb_xCuSeO$ (x = 0-0.2) samples. **a** XRD patterns. **b** The tetragonal structure (space group: P4/nmm) of BiCuSeO. **c** Nominal Pb-content dependence of the lattice parameters (a and c).

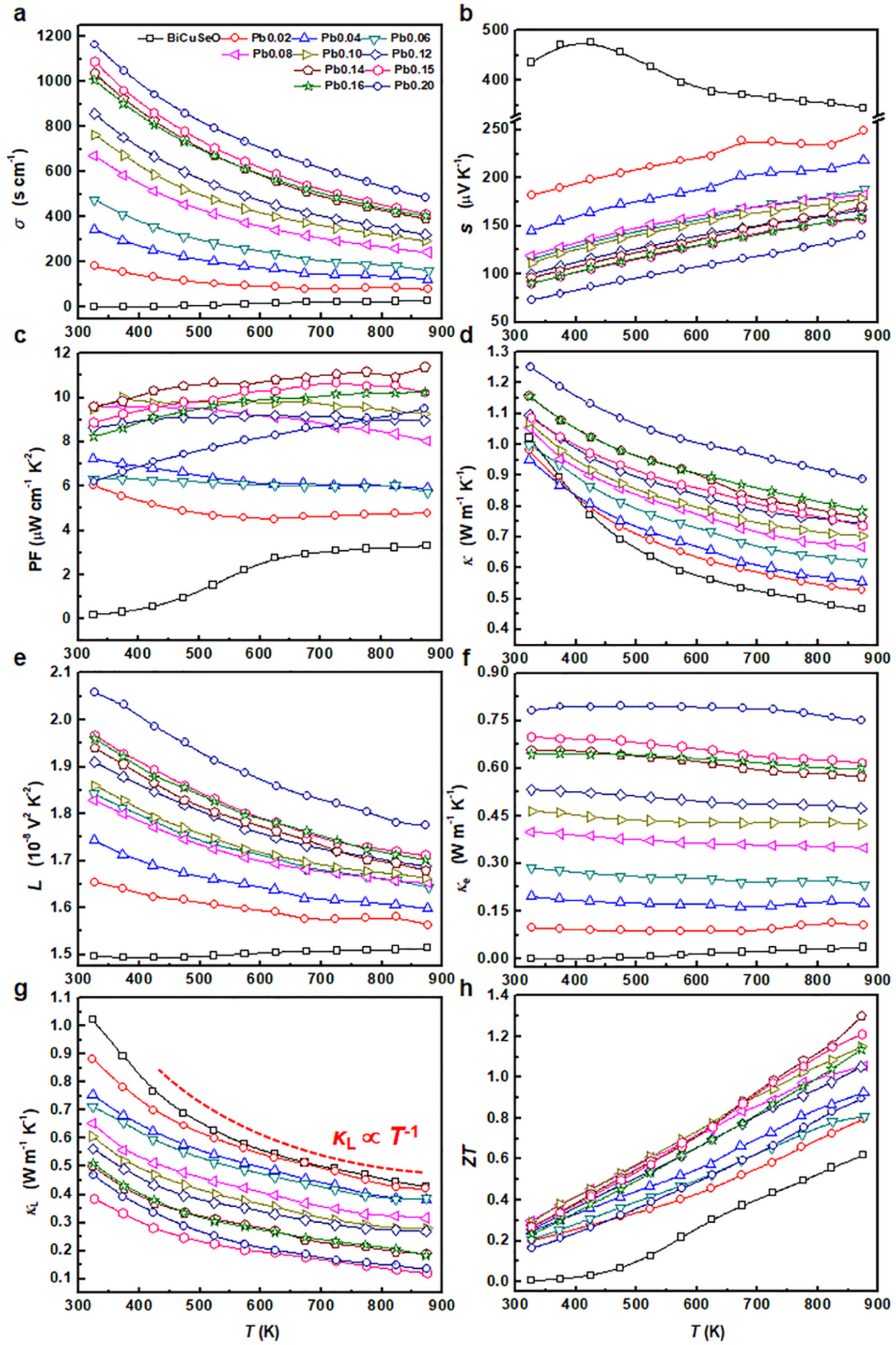

Supplementary Figure 4. Thermoelectric properties of $Bi_{1-x}Pb_xCuSeO$ (x = 0-0.2). Temperature dependences of **a** electrical conductivity, **b** Seebeck coefficient, **c** power factor, **d** total thermal conductivity, **e** Lorenz parameter, **f** electronic thermal conductivity, **g** lattice thermal conductivity, and **h** dimensionless figure of merit *ZT*.

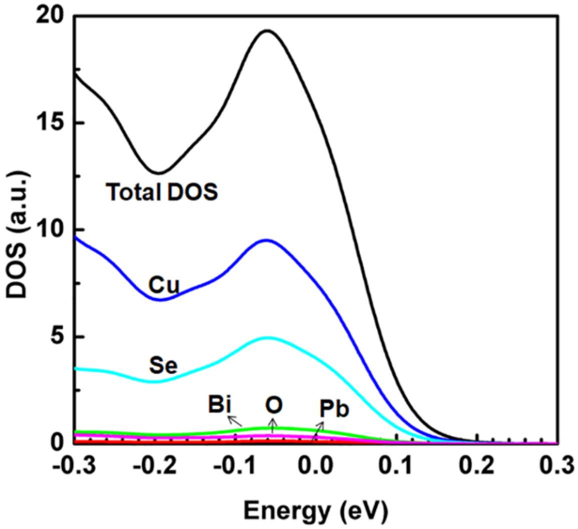

Supplementary Figure 5. The pDOS of different elements for $Bi_{0.875}Pb_{0.125}CuSeO$ at the top of valence band.

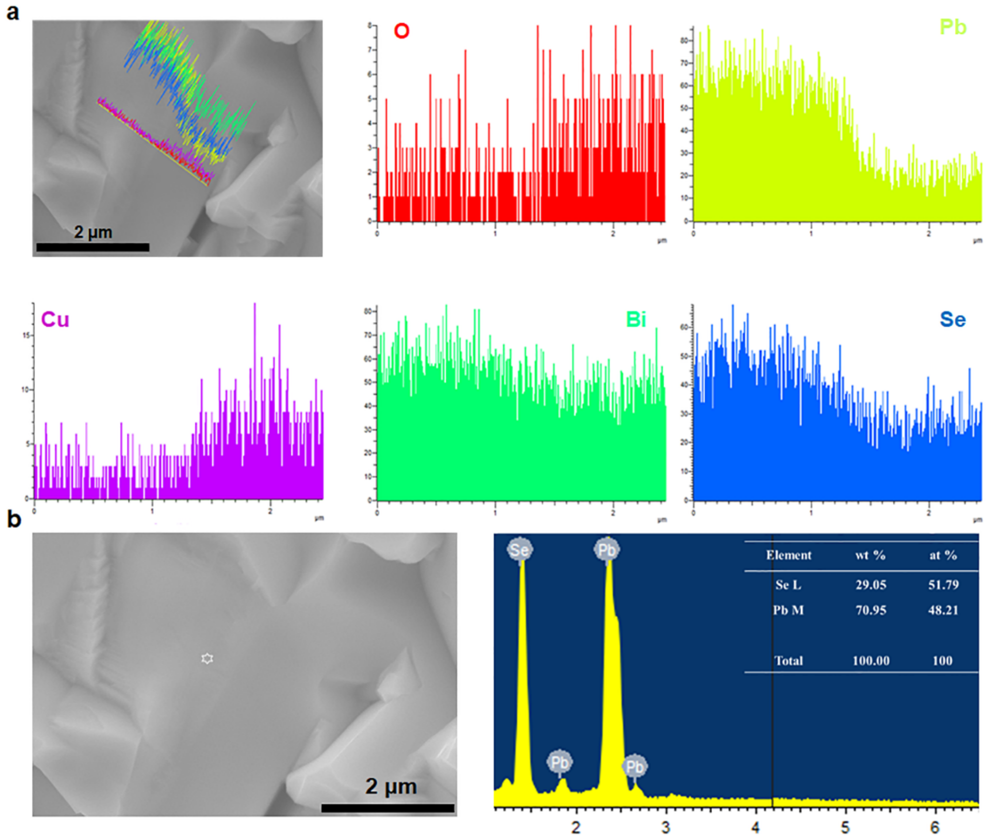

Element	wt %	at %
Se L	29.05	51.79
Pb M	70.95	48.21
Total	100.00	100

Supplementary Figure 6. Phase characterization. **a** Line scanning results for $Bi_{0.86}Pb_{0.14}CuSeO$. The interfaces between the two materials are clearly observed, which indicates that the second phase possesses more Pb and Se, as compared with those in $Bi_{1-x}Pb_xCuSeO$. **b** Energy dispersive spectroscopy **(**EDS) analysis of $Bi_{0.86}Pb_{0.14}CuSeO$, whereas p-type PbSe (Se-rich) can be clearly detected.

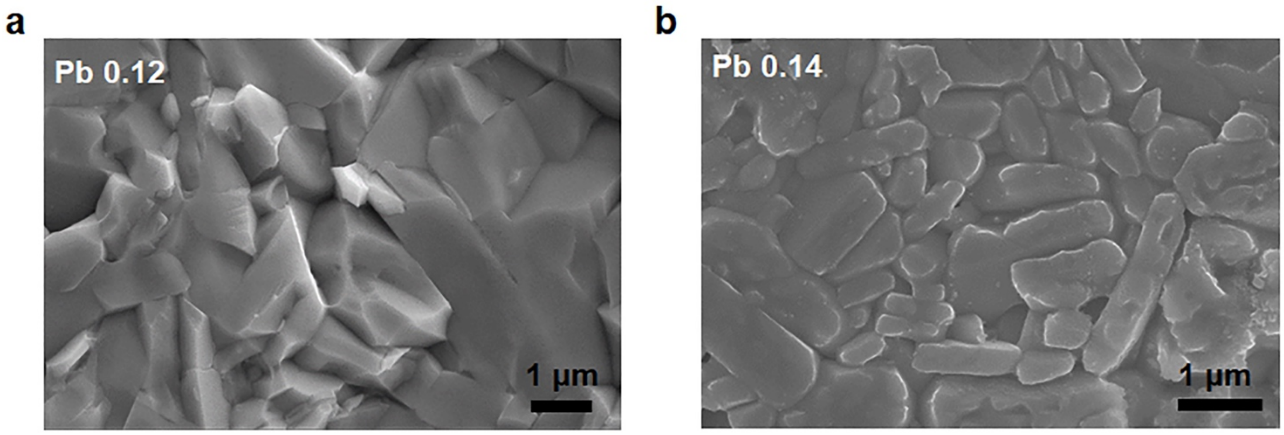

Supplementary Figure 7. Microstructural morphology (SEM) of $Bi_{1-x}Pb_xCuSeO$. The images of **a** $Bi_{0.88}Pb_{0.12}CuSeO$ and **b** $Bi_{0.86}Pb_{0.14}CuSeO$ after thermal etching.

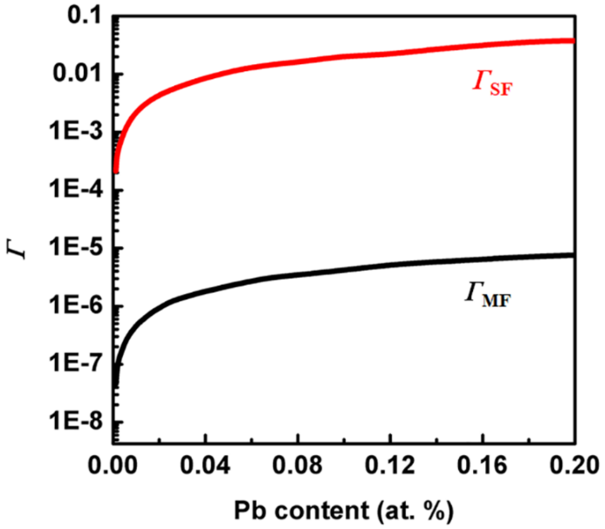

Supplementary Figure 8. Disorder scattering parameters of $Bi_{1-x}Pb_xCuSeO$ (x = 0-0.2). Comparison of the strain-field scattering parameter Γ_{SF} and mass fluctuation scattering parameter Γ_{MF}.

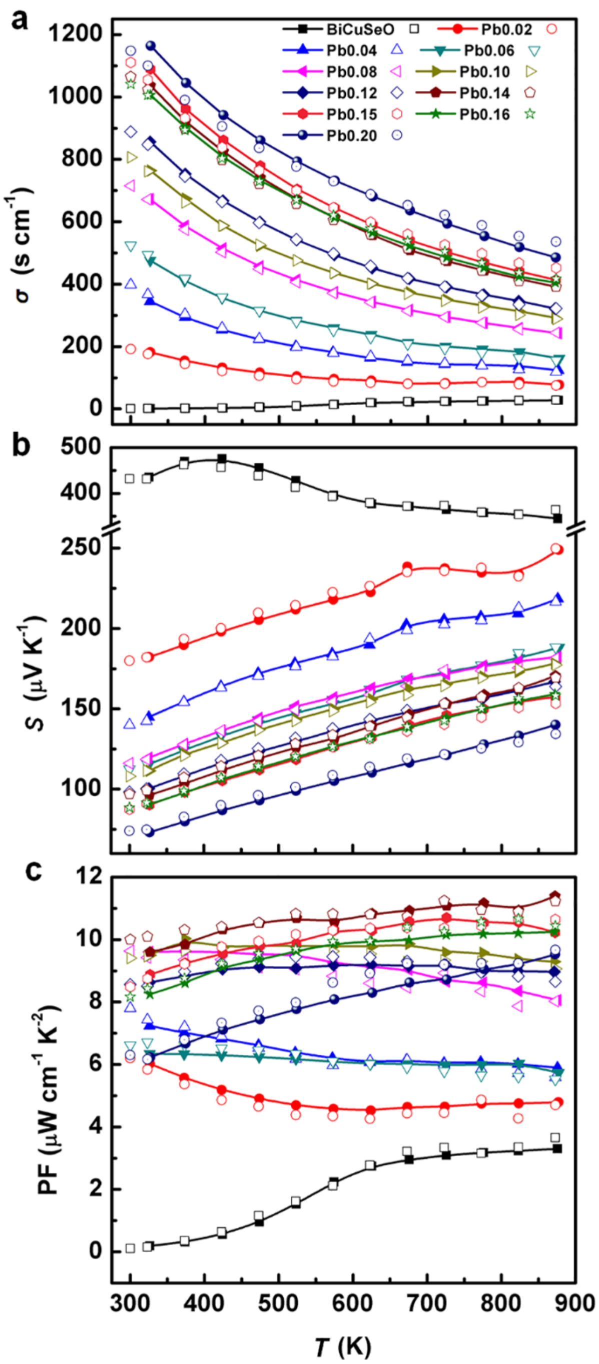

Supplementary Figure 9. Repeated measurements of the electrical properties. **a** Electrical conductivity, **b** Seebeck coefficient, **c** power factor for $Bi_{1-x}Pb_xCuSeO$ samples (x = 0-0.2) at 300-873 K. The solid symbols are indexed to the initial results (same to those of Supplementary Figure 4), whereas the open symbols are the 2nd measurements.

Supplementary Tables

Samples	p (10^{20} cm^{-3})	Ξ eV	m_d^* (m_0)	N_v	σ (S cm^{-1})	S (μV K^{-1})	PF (μW cm^{-1} K^{-2})
$x = 0$	0.02	21	0.5	0.3	2.2	310	0.2
$x = 0.02$	2.8	26	4.6	8	183	177	5.7
$x = 0.04$	5.5	26	5.3	10	339	144	7.1
$x = 0.06$	9.5	28	5.9	11	450	115	5.9
$x = 0.08$	11.5	25	6.2	12	693	108	8.1
$x = 0.10$	15.9	26	6.7	14	834	102	9
$x = 0.12$	18.9	27	6.9	14	918	92	7.8

Supplementary Table 1. The experimental data and calculated results based on the numerical modeling. Hole concentration (p), deformation potential (Ξ), effective mass (m_d^*), band degeneracy (N_v), calculated electrical conductivity (σ), Seebeck coefficient (S), and power factor (PF) values of $Bi_{1-x}Pb_xCuSeO$ (x = 0 - 0.12) at 300 K. The m_i^* used here is 1.17 m_0.

Supplementary Notes

Supplementary Note 1

Thermal diffusivity and heat capacity

Thermal diffusivity (D) was measured by a laser flash method (LFA-457, Netzsch, Germany) under a continuous Ar flow, and the data is shown in Supplementary Figure 1a.

Since specific heat C_p (Supplementary Figure 1b) would not change significantly and roughly equals to C_V (constant volume specific heat capacity) in solids, we deduced it by using the Debye model[1-3] (similar to the Dulong–Petit law when $T > \Theta_D$) and applied it for all samples. C_V can be formulated as

$$C_V = \frac{9nR}{M}\left(\frac{T}{\Theta_D}\right)^3 \int_0^{\frac{\Theta_D}{T}} \frac{x^4 e^x}{(e^x - 1)^2} \mathrm{d}x, \qquad (1)$$

where R is the ideal gas constant, n the atom number, and M the molar mass of a primitive cell.

Supplementary Note 2

Lorenz number and effective mass

To analyze the transport properties of samples, we measured the carrier concentrations and mobilities by the Hall effect. Based on the assumption of single parabolic band (SPB) model and acoustic phonon scattering, the Lorenz number (see Supplementary Figure 4) and the effective mass can be derived from the follow formulas

$$S = \pm \frac{k_B}{e}\left(\frac{(r+5/2)F_{r+3/2}(\eta)}{(r+3/2)F_{r+1/2}(\eta)} - \eta\right), \tag{2}$$

$$n = \frac{1}{2\pi^2}\left(\frac{2m^* k_B T}{\hbar^2}\right)^{3/2} F_{1/2}(\eta) \ , \tag{3}$$

$$F_i(\xi) = \int_0^\infty \frac{x^i \mathrm{d}x}{1+e^{x-\xi}}, \tag{4}$$

and $$L = (\frac{k_B}{e})^2\left(\frac{(r+7/2)F_{r+5/2}(\eta)}{(r+3/2)F_{r+3/2}(\eta)} - \left[\frac{(r+5/2)F_{r+3/2}(\eta)}{(r+3/2)F_{r+1/2}(\eta)}\right]^2\right), \tag{5}$$

where e is the elemental electron charge, F_i the Fermi integrals, and $\eta = E_F / k_B T$ the reduce Fermi energy, and E_F the Fermi energy. Meanwhile, scattering parameter r is assumed as −1/2 for the acoustic phonon scattering.[4]

Supplementary Note 3

Lattice thermal conductivity estimated by the Debye-Callaway model

The Debye-Callaway model with relaxation time approximation (RTA)[5-7] was utilized to calculated κ_L, as expressed as

$$\kappa_L = \frac{k_B}{2\pi^2 v}(\frac{k_B}{\mathrm{h}})^3 T^3 \int_0^{\frac{\Theta_D}{T}} \frac{\tau_c x^4 e^x}{(e^x-1)^2}\mathrm{d}x \ , \tag{6}$$

where $x = \hbar\omega / k_B T$, ω the phonon frequency, τ_c the total relaxation time, $\hbar$ the Planck constant, and v the mean sound velocity. The overall relaxation rate τ_c^{-1} can be determined by combining various scattering

processes as given

$$\tau_{\mathrm{c}}^{-1} = \tau_{\mathrm{N}}^{-1} + \tau_{\mathrm{b}}^{-1} + \tau_{\mathrm{d}}^{-1} + \tau_{\mathrm{U}}^{-1}, \tag{7}$$

where τ_{N}, τ_{b}, τ_{d}, and τ_{U} are the relaxation times for the Normal processes, boundary scattering, defect scattering, and the Umklapp processes, respectively. Then we can incorporate the effect of Pb point defect by modeling τ_{d}, and meso- or nano-scale effects by adjusting τ_{b}.

$$\tau_{\mathrm{d}}^{-1} = B\omega^4 = \frac{V}{4\pi v^3}\Gamma\omega^4, \tag{8}$$

Γ is the disorder scattering parameter and B is a constant that is independent of temperature and frequency, representing the mass and strain-field fluctuations.

The mass and strain-field fluctuations would scatter phonons with particular wavelengths, and their contributions to disorder scattering parameter can be given below,

$$\Gamma = \Gamma_{\mathrm{MF}} + \Gamma_{\mathrm{SF}}, \tag{9}$$

$$\Gamma_{\mathrm{MF}} = \frac{\sum_{i=1}^{n} c_i \left(\frac{\overline{M_i}}{\overline{\overline{M}}}\right)^2 f_i^1 f_i^2 \left(\frac{M_i^1 - M_i^2}{\overline{M_i}}\right)^2}{\sum_{i=1}^{n} c_i}, \tag{10}$$

$$\Gamma_{\mathrm{SF}} = \frac{\sum_{i=1}^{n} c_i \left(\frac{\overline{M_i}}{\overline{\overline{M}}}\right)^2 f_i^1 f_i^2 \varepsilon_i \left(\frac{r_i^1 - r_i^2}{\overline{r_i}}\right)^2}{\sum_{i=1}^{n} c_i}, \tag{11}$$

where c_i is the relative degeneracy of the site, f_i is the fractional occupation, $\overline{M_i}$ and $\overline{r_i}$ are the average mass and radii of that element, and $\overline{\overline{M}}$ is the average atom mass.[8] Considering the atomic masses and sizes of Bi and Pb (ionic radii $r_{\mathrm{Bi}}{}^{3+}$ = 1.17 Å and $r_{\mathrm{Pb}}{}^{2+}$ = 1.29 Å), the calculated values of Γ_{SF} and Γ_{MF} are plotted in Supplementary Figure 8. It is obvious that the strain field scattering dominates, and the mass fluctuation has a minor contribution.

Supplementary References

1 Gu, M. *et al.* Size, temperature, and bond nature dependence of elasticity and its derivatives on extensibility, Debye temperature, and heat capacity of nanostructures. *Phys. Rev. B* **75**, 125403 (2007).

2 Lazarev, V., Izotov, A., Gavrichev, K. & Shebershneva, O. Fractal model of heat capacity for substances with diamond-like structures. *Thermochim. Acta* **269**, 109-116 (1995).

3 Berggold, K. *Thermal Transport in Cuprates, Cobaltates, and Manganites* (Doctoral dissertation, Universität zu Köln, 2006).

4 Lan, J. L. *et al.* Enhanced thermoelectric properties of Pb-doped BiCuSeO ceramics. *Adv. Mater.* **25**, 5086-5090 (2013).

5 Ren, G.-K., Butt, S., Ventura, K. J., Lin, Y.-H. & Nan, C.-W. Enhanced thermoelectric properties in Pb-doped BiCuSeO oxyselenides prepared by ultrafast synthesis. *RSC Adv.* **5**, 69878-69885 (2015).

6 Callaway, J. Model for lattice thermal conductivity at low temperatures. *Phys. Rev.* **113**, 1046 (1959).

7 Callaway, J. & von Baeyer, H. C. Effect of point imperfections on lattice thermal conductivity. *Phys. Rev.* **120**, 1149 (1960).

8 Yang, J., Meisner, G. & Chen, L. Strain field fluctuation effects on lattice thermal conductivity of ZrNiSn-based thermoelectric compounds. *Appl. Phys. Lett.* **85**, 1140-1142 (2004).
